# Rapid screening and scaled manufacture of immunogenic virus-like particles in a tobacco BY-2 cell-free protein synthesis system

**DOI:** 10.3389/fimmu.2023.1088852

**Published:** 2023-01-26

**Authors:** Jorge Armero-Gimenez, Ruud Wilbers, Arjen Schots, Charles Williams, Ricarda Finnern

**Affiliations:** ^1^ Technology center, LenioBio GmbH, Dusseldorf, Germany; ^2^ Laboratory of Nematology, Wageningen University, Wageningen, Netherlands

**Keywords:** cell-free protein synthesis (CFPS), virus-like particle (VLP), vaccine, carrier VLP, hepatitis B-core antigen (HBc), tobacco BY-2, recombinant protein production

## Abstract

Several vaccine platforms have been developed to fight pathogenic threats, with Virus-Like Particles (VLPs) representing a very promising alternative to traditional platforms. VLPs trigger strong and lasting humoral and cellular immune responses with fewer safety concerns and higher stability than other platforms. The use of extensively characterized carrier VLPs modified with heterologous antigens was proposed to circumvent the viral complexity of specific viruses that could lead to poor VLP assembly and yields. Although carrier VLPs have been successfully produced in a wide variety of cell-based systems, these are limited by low protein yields and protracted clone selection and optimization workflows that limit VLP screening approaches. In response, we have demonstrated the cell-free protein synthesis (CFPS) of several variants of the hepatitis B core (HBc) carrier VLP using a high-yielding tobacco BY-2 lysate (BYL). High VLP yields in the BYL system allowed in-depth characterization of HBc variants. Insertion of heterologous sequences at the spike region of the HBc monomer proved more structurally demanding than at the N-terminus but removal of the C-terminal domain allowed higher particle flexibility and insert acceptance, albeit at the expense of thermal and chemical stability. We also proved the possibility to scale the CFPS reaction up to 1L in batch mode to produce 0.45 grams of the native HBc VLP within a 48-hour reaction window. A maximum yield of 820 µg/ml of assembled VLP particles was observed at the 100µl scale and most remarkably the CFPS reaction was successfully scaled from 50µl to 1L without any reduction in protein yield across this 20,000-fold difference in reaction volumes. We subsequently proved the immunogenicity of BYL-derived VLPs, as flow cytometry and microscopy clearly showed prompt recognition and endocytosis of fluorescently labelled VLPs by human dendritic cells. Triggering of inflammatory cytokine production in human peripheral blood mononuclear cells was also quantitated using a multiplex assay. This research establishes BYL as a tool for rapid production and microscale screening of VLP variants with subsequent manufacturing possibilities across scales, thus accelerating discovery and implementation of new vaccine candidates using carrier VLPs.

## 1 Introduction

Vaccination is the most effective method to prevent viral infections, a major cause of morbidity and early mortality in humans worldwide (1). Several vaccine platforms have emerged, from which Virus-Like Particles (VLPs) have gained increased interest over the past years. Virus-like particles (VLPs) are virus structures that lack the genetic material and molecular machinery required for viral replication. As VLPs are unable to replicate within the host, they pose none of the safety risks associated with attenuated vaccines (2). Nevertheless, given their resemblance to viruses, VLP vaccines confer strong protection against their cognate viruses by triggering robust and lasting immune responses (3–5).

Despite these advantages of VLP-based vaccines, only VLP products against Hepatitis B Virus (HBV), Hepatitis E Virus (HEV), Human Papillomavirus (HPV) and Severe acute respiratory syndrome coronavirus 2 (SARS-CoV-2) are approved for human use (6, 7). The intrinsically complex assembly mechanisms of specific viruses, like human immunodeficiency virus (8), impose lengthy proof-of-concept studies and subsequent production optimization that render virus-specific VLP vaccine candidates as non-commercially viable (9). To circumvent virus complexity, the use of carrier VLPs as vaccination platforms has been proposed. Carrier VLPs are chosen based on the availability of prior characterization data and can be decorated with antigens or peptides from different pathogens, thus exploiting the advantages of VLP platforms without their drawbacks (10).

The hepatitis B core antigen (HBc) is a non-enveloped model VLP that has been extensively studied and applied as carrier to vaccinate against various heterologous pathogens (11). The HBc VLP is composed of a 21kDa monomer that self-assembles into two different and highly stable particle conformations of either 180 or 240 monomers termed T3 and T4, respectively (12–14). The structure of the HBc VLP is pliable for modification, allowing the introduction of small external protein sequences at three different sites: N-terminal, Spike (also known as major insertion region or MIR) and C-terminal. Furthermore, protein engineering techniques have been developed to further increase carrier pliability, allowing the insertion of bigger antigens into the HBc VLP with less concerns for structural constraints (15–17). The versatility of the HBc VLP in heterologous antigen decoration thus increases its potential to be employed as a carrier VLP for vaccine candidate generation. Nevertheless, there are still production challenges that must be addressed to maximize yields and support the clinical translation of engineered HBc VLP derivatives.

Although HBc VLPs have been successfully produced in cell-based systems, these platforms commonly struggle in terms of VLP yields (18). Additionally, cell-based expression requires lengthy processes of cell culturing, genetic transformation and expression optimization, rendering rapid library screening non-viable (19). Cell-Free Protein Synthesis (CFPS) systems have been explored as increasingly viable alternatives with unique advantages. The open nature of CFPS allows reaction conditions to be modified freely to maximize protein yields, as well as allowing simpler screening (20, 21). Screening of different carrier VLP and heterologous antigen combinations could be essential to ensure proper VLP assembly and immunogenicity (22, 23). Various CFPS systems are available, derived from different prokaryotic or eukaryotic origins. Prokaryotic CFPS systems show lower production costs, higher protein yields and better production scalability than eukaryotic systems but lack the chaperones and post-translational modification machinery necessary to produce complex proteins (24, 25). Therefore, a cheaper, high-yielding eukaryotic CFPS system, capable of manufacture-scale production would be necessary to produce effective HBc-based VLP vaccines decorated with complex antigens.

**Figure 1:**
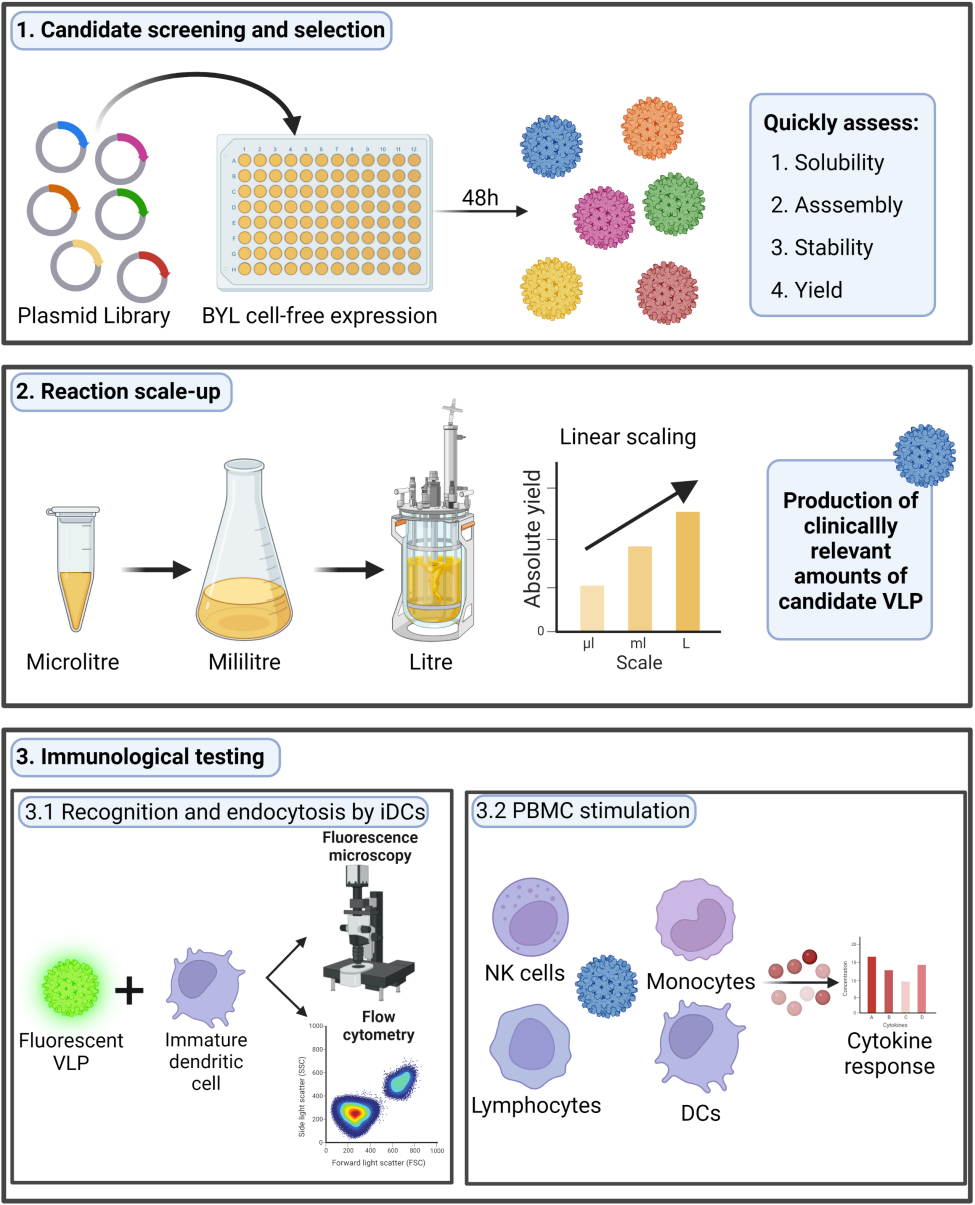
Research performed in this study. In this research we studied the possibility to use the BYL cell-free expression system to quickly screen different HBc VLP variants, scale the production and test the immunogenicity of a chosen candidate. The HBc VLP structure shown was retrieved from PDB (ID:1QGT). HBc, Hepatitis-B core; VLP, virus-like particle; BYL, BY-2 tobacco cell-free lysate; NK, natural killer; DC, dendritic cell. Figure created at biorender.com.

The BY-2 tobacco cell-free lysate (BYL) is a novel plant-based CFPS system that could serve as an effective alternative to previous eukaryotic platforms. BYL has shown higher recombinant protein production yields than other CFPS systems with the potential to cost-effectively scale both production and reaction volumes (26–28). The high yields, scalability, and possibility to produce complex proteins led to the commercialization of BYL as ALiCE^®^ (29). Nevertheless, the capability to produce VLPs in BYL has still not been shown. Moreover, although previous research proved the production of VLPs in other CFPS systems, none were scaled to provide sufficient material for follow-up immunogenicity studies with purified particles (22, 30, 31). Therefore, exploring the ability to scale the BYL reaction and determining the immunogenicity of BYL-produced VLPs will be essential if BYL is to be used as a platform for vaccine candidate screening, selection, and manufacturing in the event of a pandemic. A graphical summary of the research performed in this study is shown in **Figure 1**.

In our research, we aimed to study the production and assembly of different variants of the HBc VLP in BYL, more specifically to study the acceptability of heterologous inserts and other structural constraints related to particle modification. Overall, it was observed that BYL is a viable platform to quickly screen and produce different HBc VLP variants. Heterologous insertion at the MIR site of HBc VLPs proved more structurally demanding than at the N-terminal site. Removing the C-terminal domain of the HBc VLP positively affected the acceptance of inserts at the MIR but decreased particle stability. A maximum yield of 820 µg/ml of assembled VLP particles was observed at the 100µl scale and most remarkably the CFPS reaction was successfully scaled from 50µl to 1L without any reduction in protein yield across this 20,000-fold difference in reaction volumes. The produced VLPs were efficiently internalized by human dendritic cells and triggered strong immune inflammatory responses, observed as the release of pro-inflammatory cytokines upon human peripheral blood mononuclear cell (PBMC) stimulation. All in all, it was proven that BYL can be used to quickly screen VLP variants in terms of assembly and yield and as a tool to characterize VLP stability. Furthermore, BYL can also be used to produce immunogenic HBc VLP particles at manufacturing scale. This represents a step forward in broadening the use of VLPs for vaccination, and thus, in the fight against pathogenic threats.

## 2 Materials and methods

### 2.1 HBc VLP constructs used in this study

**Figure 2:**
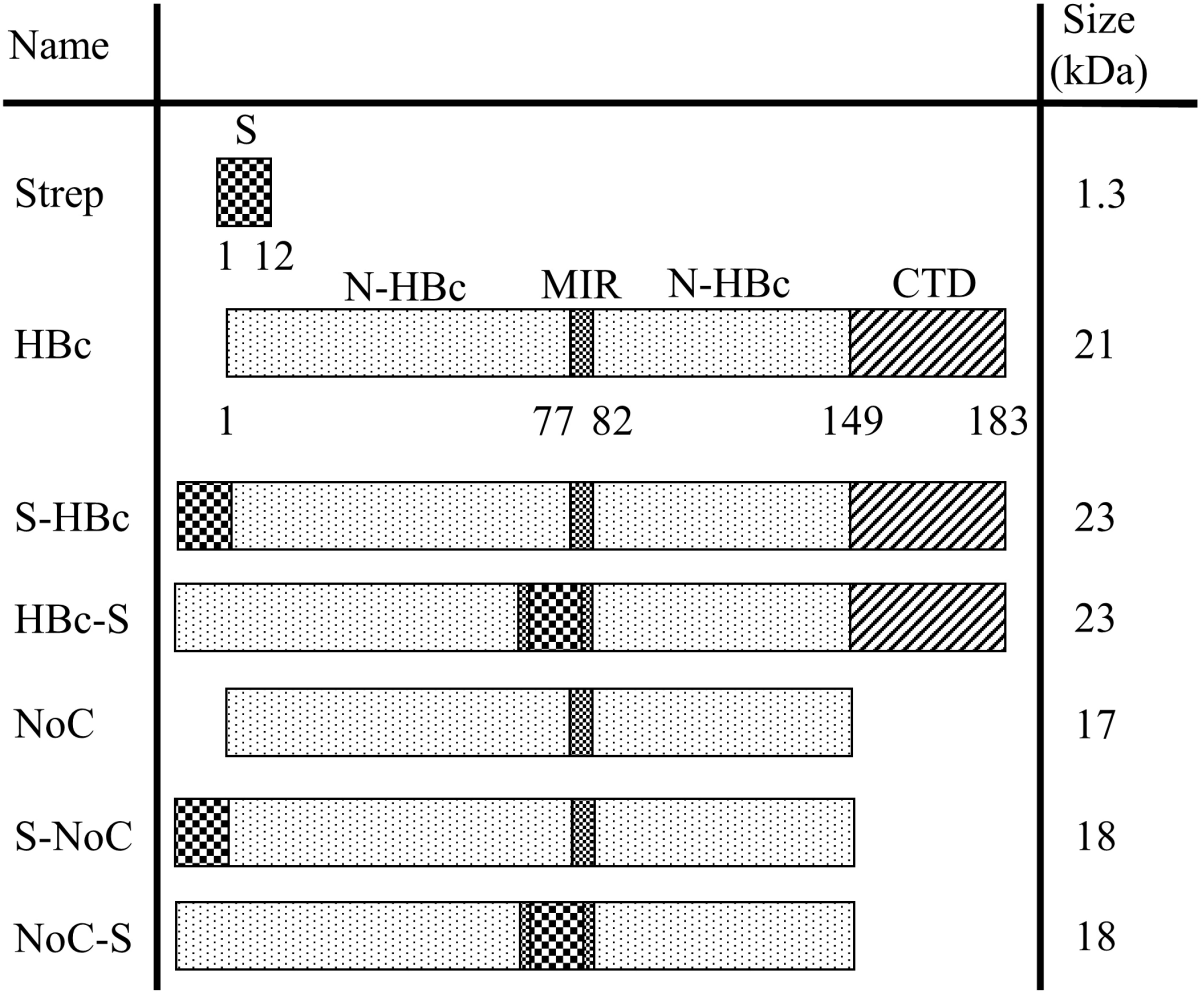
Overview of the HBc constructs used in this study. Schematic representation of the genetic constructs used to express the different HBc VLP variants in BYL. All constructs were cloned into the pALiCE01 BYL expression plasmid. The numbers under the construct indicate the amino acid position for each domain. N-HBc, N-terminal domain of the HBc monomer; MIR, major insertion region; CTD, C-terminal domain; Strep, strep-tag II insert with the GSWSHPQFEKGS sequence.

The coding sequence of the HBc VLP (*adw2* serotype) was retrieved from NCBI (GenBank: E00120.1), and codon-optimized to match codon usage in *Nicotiana tabacum* using the codon optimization tool from Integrated DNA Technologies (IDT). Six HBc VLP constructs were designed, including the full native sequence (HBc) and the HBc lacking the CTD (NoC). These two constructs were further modified by the inclusion of a Strep-tag II insert at either N-terminus or MIR regions surrounded by a single GS (GSWSHPQFEKGS), resulting in the S-HBc, HBc-S, S-NoC and NoC-S constructs (**Figure 2**). The resulting sequences were cloned into the pALiCE01 BYL expression vector *via* Gibson cloning (32). Expression plasmids were purified from *Escherichia coli* DH5alpha cultures using the NucleoBond Xtra Maxi kit (Macherey Nagel) and their correct assembly was confirmed by sequencing (Eurofins).

### 2.2 Small-scale and large-scale BYL reaction set-up

All the BYL used in this project was provided by LenioBio GmbH. Small-scale BYL CFPS reactions of 50 and 100µl were performed as suggested in published protocols (33). Lysate was thawed on ice and each expression plasmid was added to a final concentration of 5nM. Subsequently, reaction mixtures were aliquoted into half-well 96-well plates (50µl reactions) or full-well 96-well plates (100µl reactions) and incubated for 48h in a KuhnerShakerX at 25°C with 75% humidity and 500 rpm with a 12.5mm shaking diameter. The Duetz lid system (EnzyScreen BV) was used to ensure even evaporation rates along the plate. For 5, 10 and 100ml reactions, BYL supplemented with 5nM of plasmid were incubated in a KuhnerShakerX for 48h in sterile 100, 250 and 5000ml Erlenmeyer flasks, respectively, at 25°C and 95rpm with a shaking diameter of 50mm. For the 1L reactions, a Kuhner SB10 orbital shaker was used, and the supplemented lysate transferred to a Kuhner SB10 single-use bag. The one-liter lysate was then incubated at 25°C for 48h at a shaking frequency of 62rpm and 50mm shaking diameter.

### 2.3 BYL reaction analysis

After reaction completion, the lysate was collected and centrifuged at 15,000xg for 10 min to clarify the solution of insoluble lysate components. Supernatants were carefully collected, and the pellet fractions were resuspended in PBS buffer in the original lysate volume. The full-lysate, pellet and supernatant fractions could then be analyzed for VLP expression, as well as to give a preliminary view on VLP assembly. For that purpose, NuPAGE™ 4 to 12%, Bis-Tris (Invitrogen™) SDS-PAGE pre-cast gels were used. Protein samples were prepared according to the manufacturer’s instructions and a total of 1µl from each fraction was loaded onto the gel. 5µl of PageRuler ™ Prestained Protein Ladder (ThermoFisher Scientific) were also loaded as size standard. Gels were then run in 1x MES running buffer (ThermoFisher Scientific) for 25min at 200V or until sufficient resolution was obtained. Successively, gels were stained with Coomassie Blue-staining solution (25% (v/v) isopropanol, 10% (v/v) acetic acid and 0.05% (w/v) Coomassie brilliant blue R-250) and de-stained with 10% acetic acid until protein bands were clearly discernible. Gel images were captured using the Gel Doc XR+ UV transilluminator (Bio-Rad Laboratories, Inc.).

Dot blot analysis was used to give a pre-liminary view on VLP assembly using the anti-HBc antibody 3120 (Cosmo Bio). 1µl of each BYL fraction was spotted on an activated PVDF membrane and dried on a paper stack. After drying, the membrane was re-activated, and blocked for 1 hour with 5% (w/v) powder milk in PBS-T. The primary antibody was incubated at 1:5000 dilution and regular incubation and washing steps were followed. An HRP-linked Goat Anti-mouse IgG (Jackson ImmunoResearch) was used as secondary antibody at a 1:5000 dilution, and signal development was performed using the SuperSignal Chemiluminescent substrate (Thermofisher). Imaging was performed using ChemStudio Plus (Analytikjena).

### 2.4 HBc VLP purification and TEM analysis

Purification workflows began from CFPS reaction supernatant. Next, a heat treatment was applied for 1h at 70°C for the HBc VLP and its variants, and at 50°C for the NoC VLP and its variants. A centrifugation for 10 min at 15,000xg followed, after which the supernatant was collected and processed again equally for both HBc and NoC-based constructs. Hydrophobic interaction chromatography using a HiTrap Phenyl HP resin column (Cytiva) and size exclusion chromatography using a Superdex 200 column (Cytiva) were applied for further purification. All chromatographic procedures were performed on an ÄKTA purifier (Cytiva). Pure VLP samples were characterized *via* Transmission Electron Microscopy (TEM) to confirm particle assembly and integrity. Formvar-carbon coated 400 mesh copper grids (SigmaAldrich) were glow discharged in vacuum for 20 s. 10 µl of each VLP suspension was placed on a grid and incubated for 2 min. Negative staining was performed with 1% (v/v) phosphotungstic acid (PTA, pH 7.2) for 1 min. The specimens were examined in a JEOL 1400 transmission EM equipped with a Matataki (2K × 2K) camera.

### 2.5 Thermal and chemical stability analysis

To compare the thermal stability of the HBc and NoC constructs, 100µl lysate supernatant samples were incubated for 1 hour at the indicated temperatures. Afterwards, samples were centrifuged at 15,000xg for 10min, the supernatant was carefully collected, and the pellet was resuspended in PBS as to reach the original final volume. Gel protein analysis was performed as previously described. To assess chemical stability, pure HBc and NoC VLPs were precipitated by adding a saturated ammonium sulphate (AMS) to reach 40% v/v. Afterwards, the mixture was centrifuged at 4°C and 15,000xg for 30 min to drive VLP precipitation and the pellet was re-suspended in the indicated urea concentrations in 50mM phosphate buffer (pH 8.5) + 1mM DTT. After overnight incubation at 37°C, samples were run in a Superdex 200 SEC column (Cytiva). For the NoC construct, fractions containing the disassembled monomer fraction after SEC were collected and combined. For re-assembly, 200µl of the disassembled NoC monomer were dialyzed overnight against 50mM Tris (pH 7) + 800mM (NaCl). The resulting sample was then concentrated by AMS precipitation before SEC separation in PBS buffer (pH 7.4) to assess VLP re-assembly.

### 2.6 Native HBc VLP yield quantification

An in-gel quantification method was performed to assess expression yields within the lysate. 2, 1, 0.5 and 0.25 µg of Bovine Serum Albumin (BSA) were loaded on an SDS-PAGE gel alongside the samples to be quantified. Coomassie blue staining was performed as previously described and VisionWorks software was used to quantify the amount of HBc VLP on each sample given the BSA calibration curve. Background signal was subtracted using a rolling disc correction. To quantify only properly assembled HBc VLP and reduce background protein from the lysate, 100µl of each supernatant sample were heat treated at 70°C for 1 hour and then centrifuged at 15,000xg for 10 min to remove denatured protein. The supernatants from the heat treatment were used for quantifications. All scaling-up experiments were performed in triplicate except for the 1L expression.

### 2.7 Dendritic cell uptake assay

PBMCs were isolated *via* traditional Ficoll gradient (34). Myeloid immature dendritic cells (iDCs) were differentiated from monocytes purified from the isolated PBMCs by a monocyte adherence protocol (35). Briefly, PBMCs were seeded in a 6-well plate at a density of 2.5 x 10^6^/cm^2^ in 3ml of RPMI medium. Two hours after plating, non-adherent cells were removed, and adherent cells were washed three times with RPMI medium. Cells were then incubated for 6 days in RPMI medium supplemented with 20ng/ml of both human IL-4 and GM-CSF, with a medium exchange on day 3. Detached iDCs were harvested at day 6 by pipetting up and down several times. HBc VLPs were fluorescently labelled using the Alexa-488-TFP ester (ThermoFisher). Non-bound label was removed using a G25 sephadex column (Cytiva). Two micrograms of labelled VLP were incubated with 1 x 10^6^ iDCs for 2 hours for both, confocal and flow cytometry analysis. After incubation, cells were washed twice with PBS and then processed accordingly for each method. For flow cytometry studies the following marker-specific anti-human antibodies from BioLegend were used: CD14-Alexa488 (clone 63D3) and DC-SIGN-Alexa647 (clone 9E9A8). The gating strategies applied can be found in **Supplementary Figure S1**. The corresponding isotype controls were used for each antibody and fluorophore combination. The Human TruStain FcX™ Fc Receptor Blocking Solution (Biolegend) was used in all flow cytometry assays to block Fc sites and reduce non-specific signal. Flow cytometry acquisition was performed with a Cytoflex flow cytometer (Beckmann), and data was analysed with FlowJo software. For confocal microscopy, cells were seeded in a Nunc™ Lab-Tek™ Chambered Coverglass (ThermoFisher) and samples were then analyzed in a Stellaris 5 Confocal LSM (Leica).

### 2.8 PBMC stimulation assay

Native HBc VLPs produced using BYL were co-incubated with PBMCs from 6 different donors. To prevent interferences in the assay, residual endotoxins from the previously purified HBc VLP sample were removed using the EndoTrap^®^ HD 1ml prepacked columns (Lionex), and endotoxin content was measured using the Endosafe Cartridges system (Charles River). Endotoxin content for the highest protein concentration in the assay was measured to be lower than 0.2 ng/ml. After endotoxin clearance, the buffer of the samples was exchanged to PBS and sterile filtered. Isolated PBMCs were plated in a 96-well plate at 5 x 10^5^ cells/ml in 200µl RPMI 1640 medium (Thermofisher) supplemented with 10% (v/v) fetal bovine serum and 50 U/ml penicillin and 50 μg/ml streptomycin (from now own regarded as RPMI medium). Cells were then incubated 2 hours at 37°C, 5% CO_2_, after which stimulation was performed. Bacterial Lipopolysaccharide (LPS, Thermofisher) at 100ng/ml was used as a positive control for stimulation and sterile PBS was used as negative control. BYL-produced HBc VLPs were used for stimulation at 10, 1 and 0.1µg/ml and all conditions were tested in triplicate for 4 of the donors and in duplicate for 2 of them. After 24h incubation, the supernatants were collected, aliquoted and frozen at -80°C for further analysis. Cell viability after stimulation was assessed using the eBioscience Annexin V apoptosis kit (Thermofisher).

Cytokine content in the supernatants of stimulated PBMCs was measured using the LEGENDplex™ human inflammatory panel 1 kit (BioLegend), following the protocol suggested by the manufacturer (without dilution of the supernatant). The cytokine response for 13 different cytokines was measured: IL-1β, IFN-α2, IFN-γ, TNF-α, MCP-1, IL-6, IL-8, IL-10, IL-12p70, IL-17A, IL-18, IL-23, IL-33. Data was generated using a Guava-Flow flow cytometer, and the online LegendPlex software was used for analysis. A non-parametric Wilcoxon signed-rank test was performed to assess the difference in cytokine response of the LPS and VLP stimulated against the PBS negative control and each other. JMP software was used for all statistical analyses.

## 3 Results

### 3.1 Assembly screening of HBc VLP variants in BYL reveals enhanced insert acceptance upon C-terminal domain removal

Six different VLP constructs were designed to assess both the capabilities of BYL to produce functional HBc VLPs and the effect of removing the C-Terminal Domain (CTD) in particle assembly and insert acceptability. A heterologous insert was designed using a Strep tag II surrounded by a glycine and serine residues on each side. This insert was included at the N-terminus and in the MIR site of the HBc monomer, with and without the CTD (**Figure 2**). The resulting HBc VLP coding sequences were cloned into the pALiCE01 vector and expressed in BYL. VLP expression and solubility before and after centrifugation was analyzed by gel electrophoresis. To assess assembly, a dot blot assay using an antibody against a structural epitope of the HBc VLP and ultimately Transmission Electron Microscopy (TEM) were used.

**Figure 3:**
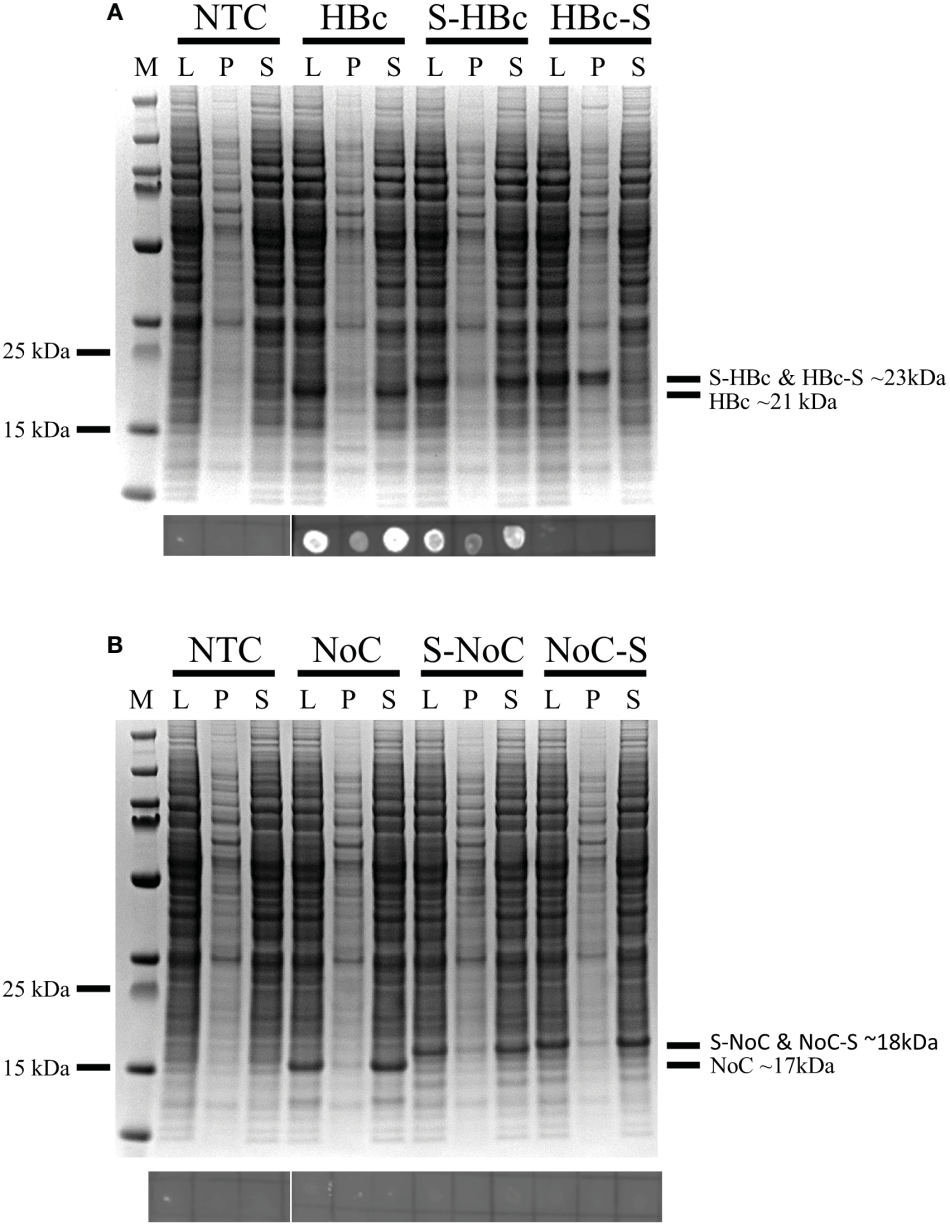
Expression and assembly analysis of the different HBc VLP constructs in BYL. SDS-PAGE (upper panels) and dot-blot (lower panels) results for the different HBc constructs after expression in BYL. For the dot blot, the conformational antibody 3120 was used, binding only to properly assembled particles **(A)** Expression and assembly analysis for the HBc constructs **(B)** Expression and assembly analysis for the NoC constructs. NTC, non-template control; HBc, hepatitis-B core antigen; L, lysate after reaction P, pellet fraction after lysate centrifugation S, supernatant fraction after lysate centrifugation; NoC, HBc core antigen lacking the C-terminal domain; S, Strep-tag II insert, located either at N-terminus (S-) or spike (-S) of the HBc and NoC constructs.

**Figure 4:**
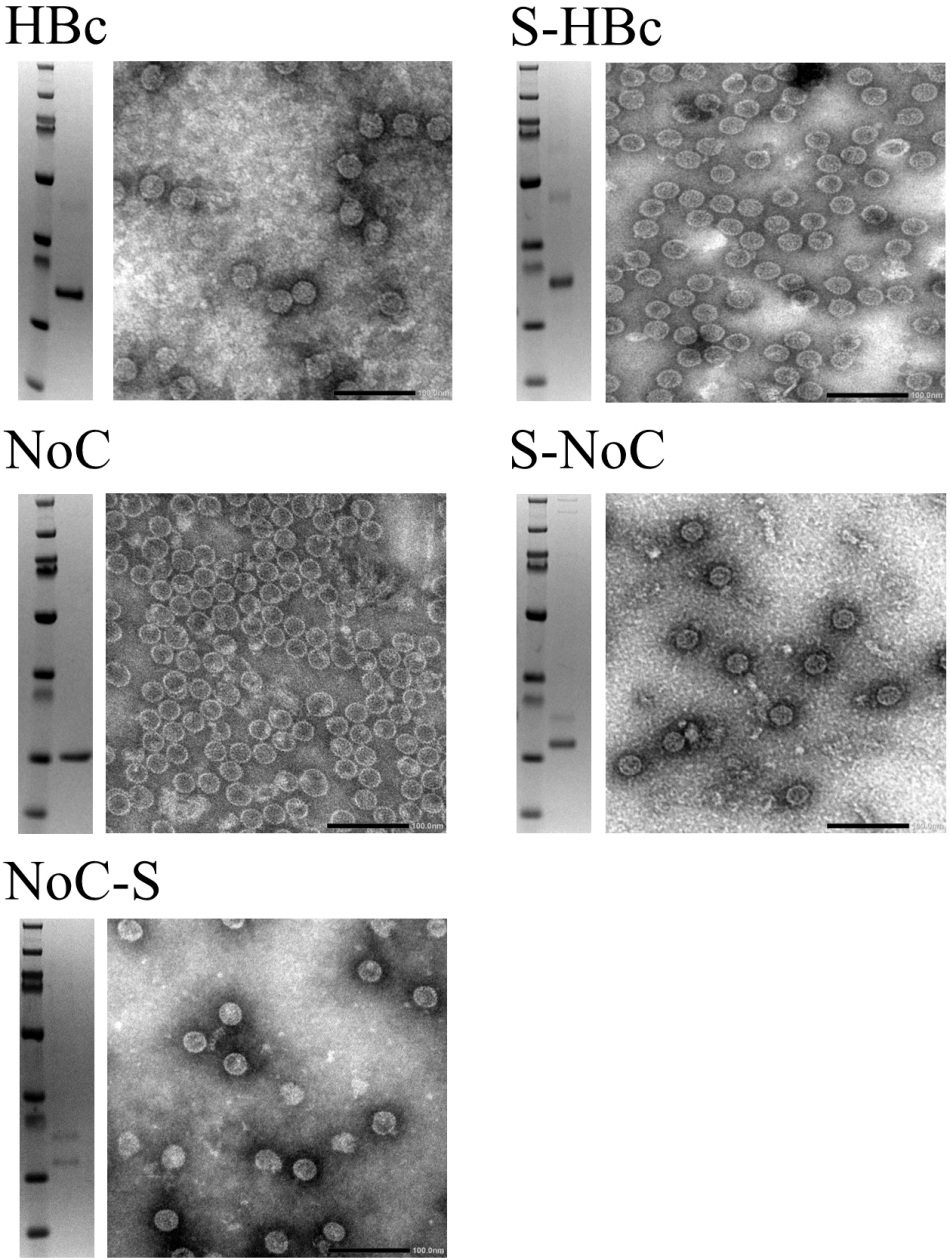
Transmission electron microscopy of HBc VLP constructs produced in BYL. The SDS-PAGE result after purification is shown on the left of each panel, and the corresponding TEM to the right for the different HBc VLP constructs. HBc, hepatitis-B core antigen; NoC, HBc core antigen lacking the C-terminal domain; S, Strep insert, located either at N-terminus or spike of the HBc and NoC constructs.

From the expression studies, high protein yields were observed for all the different constructs at the expected sizes: 23 and 21kDa for the Strep-fused and native HBc VLP constructs respectively, and 18 and 17kDa for the respective VLP constructs lacking the CTD (**Figure 3**). From all the constructs expressed, only the full-length HBc with the Strep tag II insert at the MIR site showed monomers in the non-soluble fraction, indicating misfolding and aggregation (**Figure 3A**). In the dot blot assay, a clear signal was observed for the native and N-terminally fused HBc constructs, hinting at proper particle assembly. A weaker signal was also observed in the non-soluble pellet fraction for both constructs. No signal was observed for the MIR-fused HBc construct, nor for any of the VLP constructs lacking the CTD (**Figure 3B**). Dot-blot signal was recovered for the particles lacking the CTD upon disassembly and re-assembly (**Figure S2**). VLP formation was observed in TEM analysis for all the designed constructs, except for the HBc with Strep at MIR (**Figure 4**). Overall, BYL can be employed to quickly produce and analyze the assembly of different HBc VLP constructs. Furthermore, removing the CTD of the HBc VLP has a positive effect in insert acceptability at the MIR site but prevents direct dot blot analysis.

### 3.2 Removing the C-terminal domain from HBc VLPs decreases chemical and thermal stability

**Figure 5:**
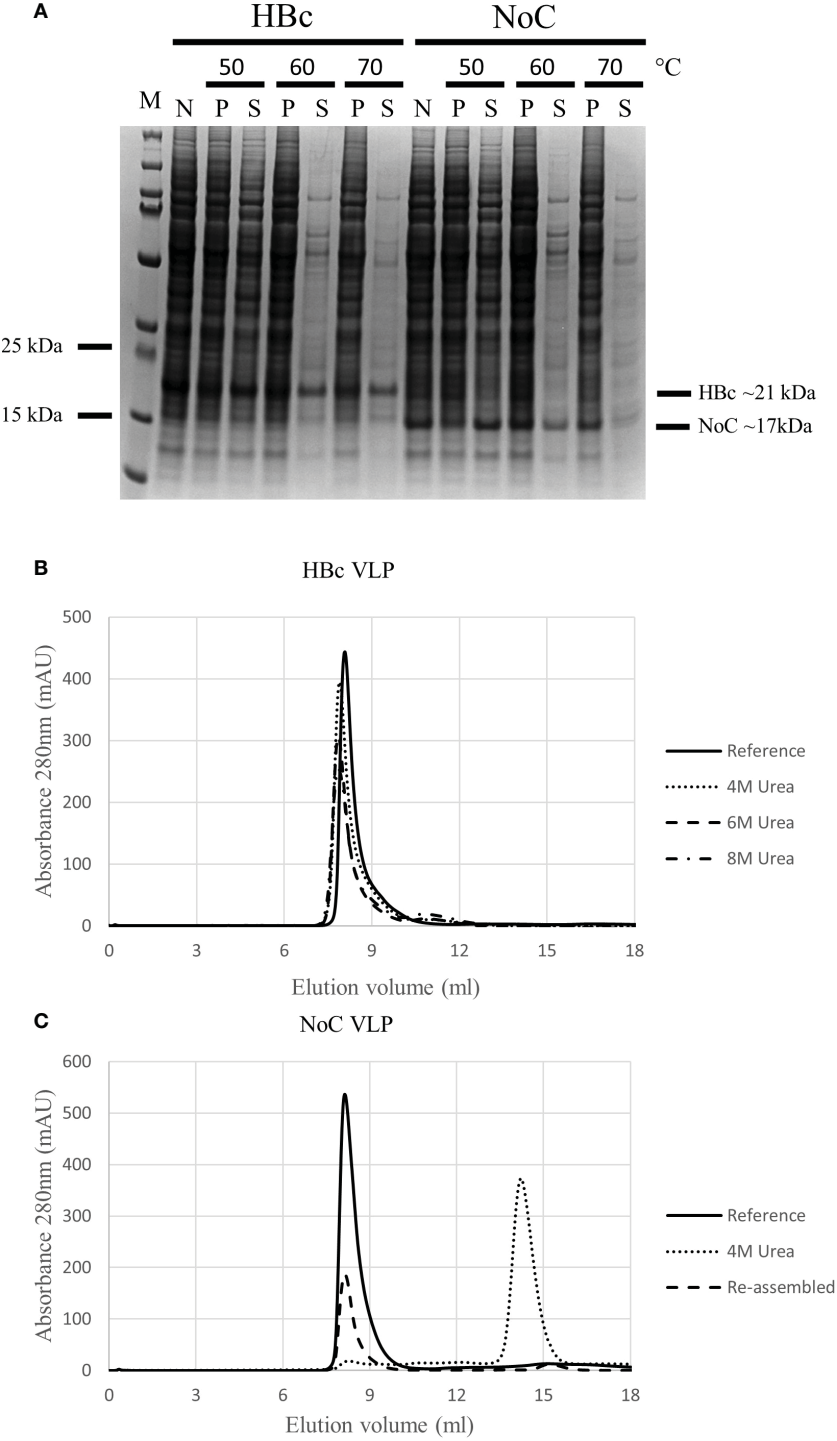
Thermal and chemical stability of HBc and NoC VLP particles produced in BYL. **(A)** Differences in thermal particle stability upon removal of the CTD as shown with SDS-PAGE results after 1 hour heat treatment of HBc and NoC VLP particles at the indicated temperatures. **(B)** Differences in chemical particle stability as shown using size exclusion chromatography of HBc VLP particles treated with the indicated urea concentrations and 1mM DTT. Reference indicates behavior for un-treated HBc VLP **(C)** Disassembly of NoC VLP particles treated with 4M urea and 1mM DTT and re-assembled in 50mM Tris-HCl (pH 7) + 800mM NaCl as shown by size exclusion chromatography. M, molecular weight marker; N, non-heat-treated; P, pellet after heat treatment; S, supernatant after heat treatment; HBc, hepatitis-B core antigen; NoC, HBc core antigen lacking the C-terminal domain; S, Strep insert, located either at N-terminus or spike of the HBc and NoC constructs.

We hypothesized that the increased insert acceptance on the HBc VLP upon CTD removal was triggered by an increase in particle flexibility, which could in turn have a negative effect on VLP stability. As VLP stability is an important factor in vaccine production, the thermal and chemical stability of the HBc VLP with and without the CTD were assessed. Thermal stability was assayed by incubating the VLP samples for 1 hour at 50°C, 60°C and 70°C. Chemical stability was assessed by incubating the VLPs with increasing concentrations of urea from 4M to 8M, with disassembly determined by analytical Size Exclusion Chromatography (SEC). HBc VLPs produced in BYL showed high thermal stability, remaining soluble after heat treatment at 70°C without detectable loss due to denaturation (**Figure 5A**). The removal of the CTD reduced VLP thermal stability, observed as protein loss at temperatures above 50°C. Removal of the CTD also had a negative effect on chemical stability, as this VLP construct promptly disassembled at 4M urea whilst the native HBc particle could not be disassembled even when 8M urea was used (**Figures 5B, C**). The monomers lacking the CTD could be re-assembled upon buffer exchange (**Figure 5B**), forming properly assembled particles (**Figure S2**). In conclusion, CTD removal increases the flexibility of the HBc VLP particle, at the expense of a reduction in thermal and chemical stability.

### 3.3 Scaled manufacture of HBc VLPs using BYL

**Figure 6:**
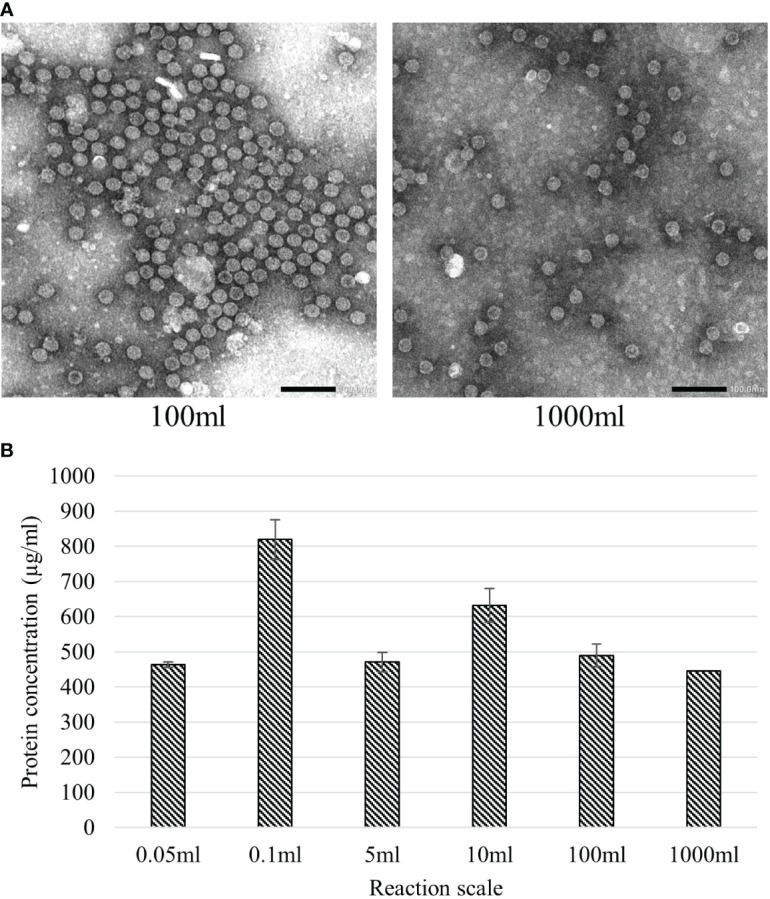
Effect of upscaling the BYL reaction volume on HBc VLP assembly and expression yield. **(A)** Transmission electron microscopy pictures of HBc VLPs produced at different scales in BYL **(B)** Calculated average of HBc VLP yields at the different BYL reaction scales. All expressions were performed in triplicate, except the 1000ml scale. HBc, hepatitis-B core antigen VLP.

The native HBc VLP construct was chosen to examine the possibilities to scale-up the BYL reaction. More specifically, the effect of reaction scale on yield and particle assembly was studied. For this purpose, the reaction was performed in six increasing scales: 0.05, 0.1, 5, 10, 100 and 1000 ml, spanning a 20,000-fold range of CFPS reaction volumes across microplate, Erlenmeyer flask and bioreactor vessels. Post-expression, samples from the different reactions were heat-treated at 70°C for 1 hour to eliminate background proteins and improperly assembled HBc protein that could interfere with yield measurements. No discernible effect on particle assembly from increasing the production scales was observed under TEM (**Figure 6A**). Linear scaling was observed with nearly identical HBc VLP yields of around 450 µg/mL at 0.05, 5, 100 and 1000 mL BYL CFPS reaction volumes (**Figure 6B**). Higher yields of 820 and 630 µg/mL were obtained at the 0.1 and 10 mL scales, respectively, suggesting that further optimization of reaction conditions is possible to maximize production at larger scales. All in all, it was shown that BYL can reach considerably high expression yields for the HBc VLP, and that the reaction is scalable up to 1L without a discernible effect in particle assembly or yield, resulting in 445mg of purified VLP material from the 1L reaction.

### 3.4 BYL-produced VLPs trigger a robust innate immune response

**Figure 7:**
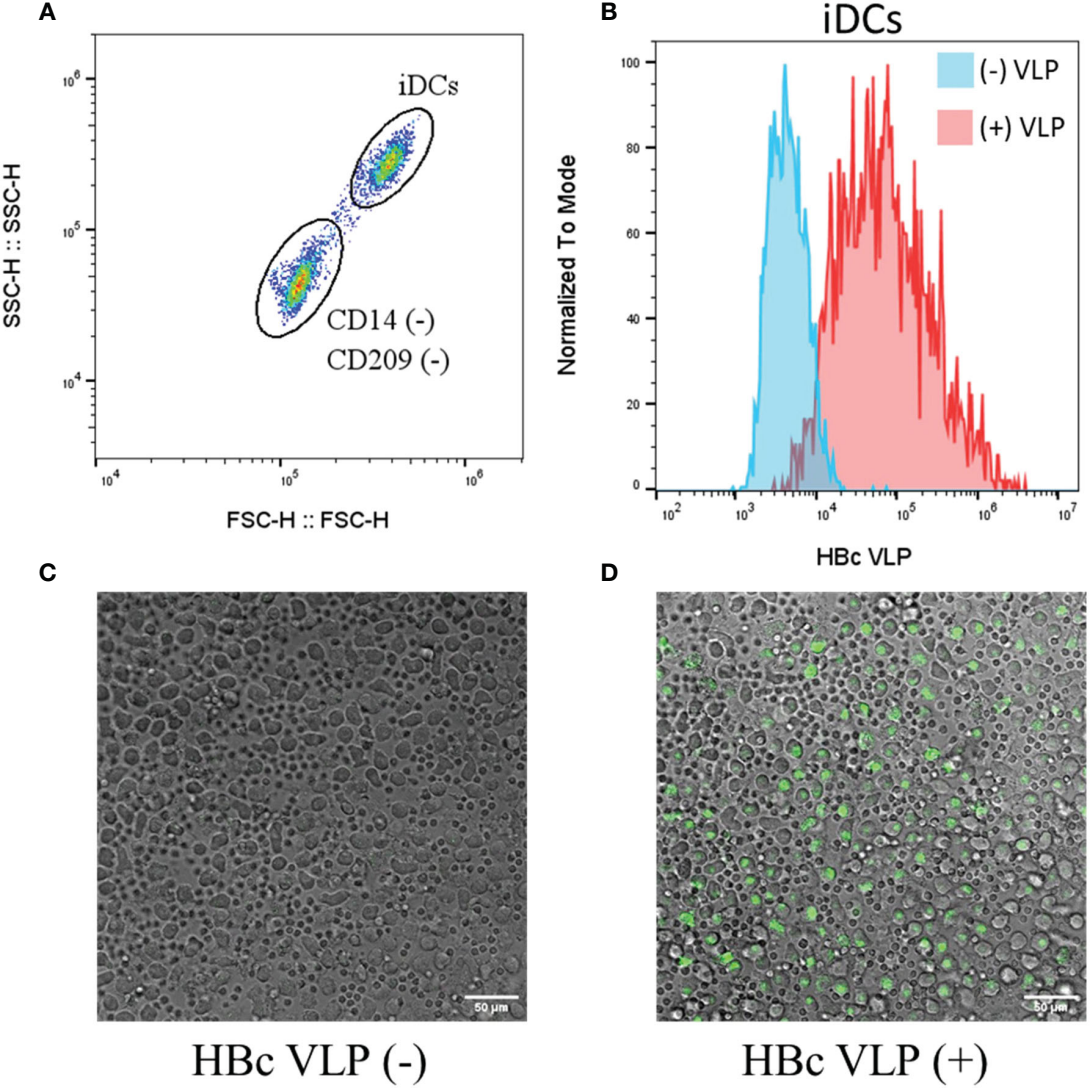
Recognition and endocytosis of fluorescently labelled HBc VLPs by human iDCs. Human iDCs were differentiated from isolated monocytes and incubated with a fluorescently labelled HBc VLP **(A)** Flow cytometry analysis of the cells after iDC differentiation, showing the two cell populations obtained **(B)** Flow cytometry analysis for the iDCs non-incubated (blue) and incubated (red) with fluorescent HBc VLPs **(C)** Fluorescent confocal imaging shows the composite fields of non-incubated cells and **(D)** fluorescent HBc VLP-incubated cells. HBc, hepatitis-B core antigen VLP; FSC, forward scatter; SSC, Side scatter; iDCs, immature dendritic cells.

To test whether the BYL-produced HBc VLPs can be recognized by the human immune system, fluorescently labelled HBc VLPs purified from the 100ml reaction were co-incubated with human immature dendritic cells (iDCs). Flow cytometry and confocal microscopy were used to determine whether the VLPs were recognized and endocytosed by the iDCs. Two cell populations were observed by flow cytometry after iDC differentiation: A CD209 (+) and CD14 (–) population that corresponded with the differentiated iDCs and an unidentified CD14 (–) and CD209 (-) population (**Figures S1, 7A**). iDCs showed a clear shift in fluorescence after incubation with the fluorescent VLP, indicating their recognition (**Figure 7B**). The unidentified cells were also shown to interact with the fluorescent VLP, but the increase in median fluorescence was 5x lower than for the iDCs (data not shown). Confocal imaging shows endocytosis by the bigger iDCs whereas the smaller unidentified cell population in the sample remained unstained (**Figure 7C, D**). Therefore, these results indicate that the HBc VLPs produced in ALiCE^®^ are efficiently recognized and endocytosed by human iDCs.

**Figure 8:**
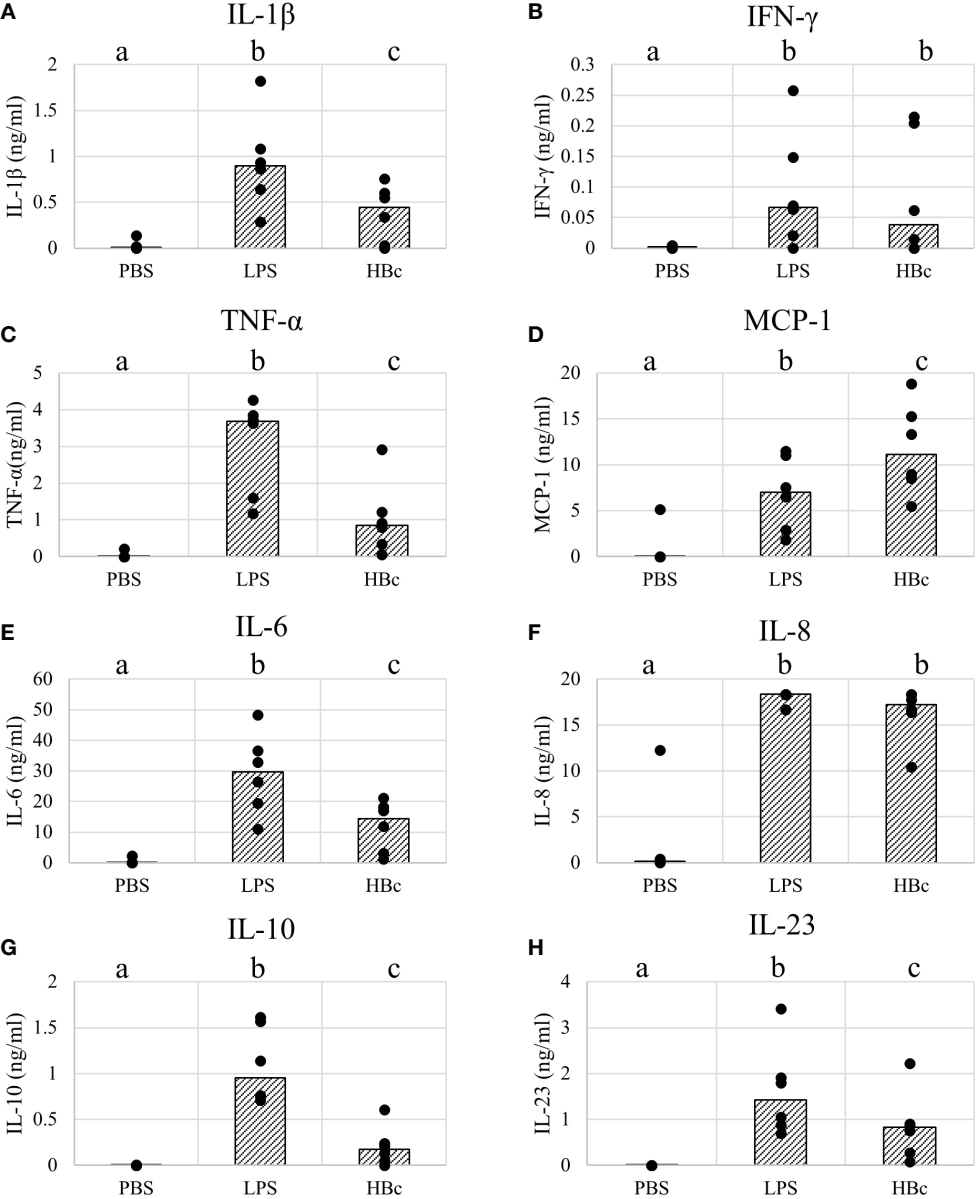
Cytokine responses of PBMCs stimulated with HBc VLPs produced in BYL. Cytokines were measured from supernatants of 6 different human blood donors stimulated for 24h with either PBS, 100ng/ml of LPS or 10µg/ml of HBc VLP. The average for each donor (dots) and the median of all donor measurements (bars) are shown for the measured cytokines: **(A)** Il-1β **(B)** IFN-γ **(C)** TNF-α **(D)** MCP-1 **(E)** IL-6 **(F)** IL-8 **(G)** IL-10 **(H)** IL-23. Different letters indicate statistically significant differences between the average of each cytokine concentration for each treatment: PBS, LPS and HBc-treated PBMCs (p<0.01, Wilcoxon signed-rank test). HBc, hepatitis-B core antigen VLP; LPS, lipopolysaccharide; PBS, Phosphate-buffered saline buffer.

Next, we aimed to determine whether a pro-inflammatory immune response would be triggered by immune cells upon VLP recognition. We stimulated human peripheral blood mononuclear cells (PBMCs) *in vitro* with the BYL-produced VLPs. VLP immunogenicity was then determined by measuring cytokine release 24 hours after VLP stimulation. Bacterial lipopolysaccharide (LPS) and PBS were used as positive and negative controls, respectively. Cytotoxic effects of the stimulation were assessed *via* propidium iodide and annexin V staining, showing no difference in cell viability compared to the PBS control 24h after stimulation (**Table S1**). Significant cytokine production was observed for the highest VLP concentration (10µg/ml) for the cytokines IL-1β, IFN-γ, TNF-α, MCP-1, IL-6, IL-8, IL-10, and IL-23 (**Figure 8**). The response level for the pro-inflammatory cytokines was in all cases lower than the positive LPS control, except for MCP-1, for which higher cytokine levels were observed for VLP-stimulated PBMCs. Also, lower levels of anti-inflammatory IL-10 were observed for the VLP-stimulated cells than for the LPS control, indicating a lower anti-inflammatory response. In the case of IL-8, most samples were at the upper detection limit (18ng/ml). Together, these data indicate that BYL-produced VLPs are both, effectively recognized by the immune system and capable of triggering a pro-inflammatory immune response *in vitro.*

## 4 Discussion

In this study, we successfully produced and studied the assembly and stability of several HBc VLP variants using the BYL CFPS system. The BYL CFPS reaction was effectively scaled up to 1 liter to produce 0.4 grams of native HBc VLP from a single 48-hour reaction, representing the largest ever CFPS reaction to produce a multimeric protein nanoparticle from either a eukaryotic or prokaryotic CFPS system. We also showed the *in vitro* immunogenic capabilities of the BYL-produced HBc VLPs, in terms of uptake by dendritic cells and cytokine response in PBMCs upon VLP stimulation. Together, these results proof the validity of the BYL system not only as a tool for fundamental research of VLP assembly, but also to support rapid candidate screening, production of clinically relevant amounts of a VLP vaccine candidate and lastly immunological testing (**Figure 1**).

The HBc VLP is a very promising platform for decoration with heterologous antigens as its structure, assembly dynamics, stability and structural constraints have been extensively studied (13, 36). The insertion site is of great importance for the immunogenicity of the insert, as it influences the presentation of antigens to the immune cells and thus, their immunogenicity (11). The MIR insertion is the most immunogenic, followed by the N-terminal insertion, and C-terminus being the least immunodominant (17, 23). We therefore aimed to determine the possibilities of producing different HBc VLP variants in BYL by designing 6 constructs with a Strep-tag II insert in the N-terminus or MIR insertion sites, with and without the CTD. Of the constructs designed, only the MIR-fused Strep-tag construct did not yield any assembled particles, whereas the equivalent construct without the CTD readily assembled into VLPs. These results indicate that the MIR region is more structurally demanding than the N-terminus regarding insertion and that the removal of the CTD allows the HBc VLP to accept the designed insert at the MIR. The structural constraints of the HBc VLP at the MIR are well studied, and several approaches have been developed to circumvent them, like splitting the monomer into two (Split core) or fusing two monomers (Tandem-core) (15, 16). Nevertheless, the mechanism by which the removal of the CTD allowed the MIR insertion in our research is unclear. This phenomenon could be caused by a decrease in the rigidness of the particle, translating to a more lenient VLP conformation, which is more amenable to modifications. In previous research, it was observed that when using tandem particles without the CTD and with two influenza inserts produced asymmetric particles, potentially indicative of this increased particle flexibility (22).

We hypothesized that the increase in particle flexibility upon CTD removal would have an effect in particle stability. Therefore, we studied the thermal and chemical stability of the native HBc particle against the one lacking the CTD. HBc VLPs without the CTD showed a lower thermal stability, with protein loss when temperatures higher than 50°C were applied for 1 hour. Furthermore, urea and DTT promptly disassembled the VLP without CTD into its monomers, that could then be re-assembled *in vitro*. These results are in line with previous research, where it was shown that the HBc particle lacking the CTD can withstand temperatures up to 70°C for 15 min without discernible VLP denaturation (37). Another study also suggests that the particle should be stable for one hour at 70°C (38). Native HBc particles showed a higher thermal resilience and could not be disassembled using urea even at the highest concentrations. In previous research, it was shown that 4M urea was enough to disassemble the full length HBc VLP produced in *E.coli* (39). This could indicate that the native HBc VLPs produced in BYL are somehow more stable than the ones produced in *E. coli*, which would be a desirable trait for a putative vaccine candidate.

Another noteworthy difference we observed between the native and the HBc VLPs lacking the CTD was the absence of signal in the dot-blot assay. We used a pre-established dot blot assay to determine VLP assembly prior to electron microscopy. The antibody 3120 was characterized to bind the capsid floor of the HBc VLP where 5 different monomers join together, thus being able to differentiate between assembled and non-assembled monomers (40, 41). In the case of the full HBc constructs, the signal correlated with the presence of assembled particles. A weaker signal was also observed in the non-soluble fraction, which could correlate with partially folded (but non-soluble) VLPs. Strikingly, no signal was observed for the constructs lacking the CTD, although assembled particles were found. The same antibody was used successfully for HBc VLPs lacking the CTD in previous research (37). A possible explanation could be the difference in purification process, as the antibody was always used in previous studies after disassembly and re-assembly of the VLP. Therefore, we performed the same dot blot assay including a disassembled and re-assembled HBc VLP without the CTD, and we observed that signal was recovered upon VLP re-assembly (**Figure S2**). This would indicate that the particles before and after reassembly are structurally different, at least regarding to the epitope that the 3120 antibody binds, although no structural differences were observed by TEM. Furthermore, the particles after assembly would be more structurally similar to the native HBc VLP, as it can bind the 3120 antibody without the need of disassembly and re-assembly. Together, these results would indicate that the dot-blot assay is a useful technique to assess assembly directly in the lysate only when using HBc VLP constructs containing the CTD.

Returning to the broader context of the CFPS reaction, a clear distinction is made in lysate origin between eukaryotic and prokaryotic systems. Eukaryotic systems can produce complex proteins that require meticulous folding or posttranslational modifications that the prokaryotic systems cannot provide. Nevertheless, this is at the expense of protein yields and lysate production and reaction scalabilities at which prokaryotic systems excel (24, 25). In our research we have shown the scaling of the BYL reaction in batch mode up to 1 liter for VLP production. To our knowledge, BYL is the only eukaryotic CFPS to reach these scales. The only other eukaryotic CFPS scaled above the microliter scale so far is the wheat-germ extract, which requires a complex discontinuous batch reaction reaching up to 10ml in volume (42). Reaching greater reaction scales would allow BYL to be used as a tool not only for VLP candidate screening, but also to quickly and economically produce the material needed for pre-liminary vaccination studies (43). Remarkably linear scaling of the BYL reaction from 0.05 to 1000 mL was observed with consistent yields of around 450 µg/mL across this 20,000-fold difference in CFPS reaction volumes. HBc VLP yields obtained in BYL are comparable to a previously reported *E. coli-*based CFPS (436 µg/L) (31) and considerably higher than an eukaryotic *Pichia pastoris-*based CFPS (6.4 µg/mL) (30) and an optimized *E. coli* cell-based system (3.2 µg/ml) (18). The BYL was previously reported to reach up to 3mg/ml of a recombinant reporter protein, indicating that further optimizations could be applied to further increment HBc VLP yields (33). Considering a reference dosage of 3.75µg from the SARS−CoV−2 VLP vaccine trial, this roughly equates to 100,000 doses that were produced in a 48-hour reaction in a single liter footprint of CFPS reaction, cementing BYL as a suitable system for rapid manufacturing scale production of VLP vaccine candidates.

VLPs are a promising platform for vaccination, given their capacity to induce strong humoral and cellular responses. For these reasons, several VLP-based vaccines have been approved for human use, and many others are under development (6). In particular, the HBc VLP model has been widely used to increment the immunogenicity of peptides from different pathogens, including malaria (44), influenza (45), *Toxoplasma gondii* (46) and many others (11). Hepatitis B core-based vaccines have proven to induce high titers of neutralizing antibodies, along with T-helper and cytotoxic responses (46–48). The production of the HBc VLPs in cell-free systems has been proven, but their immunogenicity and thus their applicability as putative vaccines has never been tested. Our research thus fills the gap from CFPS of VLPs to immunogenicity. Our results indicate that the BYL-produced HBc VLPs are efficiently recognized and endocytosed by human dendritic cells (DC) *in vitro*. The recognition and endocytosis of the VLPs by human iDCs indicate that the first steps in initiating an adaptive response are being taken, which is a mandatory requirement for any vaccine (49, 50). Furthermore, we proved that BYL-VLPs can induce a pro-inflammatory immune response in human PBMCs, inducing the production of pro-inflammatory cytokines such as IL-1β, TNF-α, IFN-γ, IL-6, IL-8 and IL-23. Interestingly, we observed a higher expression of monocyte chemoattractant protein-1 (MCP-1) than with the LPS positive control. MCP-1 is a chemokine that is produced by a wide diversity of cell-types, including macrophages and neutrophils, although non-myeloid cells have also been proven to be major mediators in MCP-1 responses (51, 52). MCP-1 mediates the recruitment of not only monocytes and macrophages, but also dendritic cells and T-lymphocytes into the inflammation site (53–55). Thus, a higher level of MCP-1 could indicate an increased recruitment of the APCs and lymphocytes required to elicit an adaptive immune response. *In vivo*, MCP-1 can lead to a Th1 or Th2 response, depending on other factors, such as the type of pathogen (56). Given our cytokine panel, the production of the IFN-γ and TNF-α could be indicative of a Th1 response, whereas the synthesis of IL-6 and IL-23 could indicate a Th17 response. Given that IL-17 was not produced in significant amounts upon stimulation, a Th17 response is unlikely. Further longer-term studies in animals are required to determine more specifically what kind of immune response is elicited, as well to study humoral responses. Altogether, these data indicate the immunogenicity of BYL-produced HBc VLPs, thus facilitating their implementation as carrier VLPs for heterologous antigens and ultimately to produce vaccine candidates in BYL.

All in all, our research sets the ground to utilize BYL as a system to rapidly study the assembly and stability of VLPs. The synthesis speed, the capability of scaling-up the VLP synthesis reaction together with their proven immunogenicity make BYL a promising platform to produce VLPs of clinical relevance. Combining these characteristics together with the decoration of carrier VLPs with antigens of interest poses an encouraging route to quickly develop vaccine candidates against new or re-emerging diseases.

## Data availability statement

The original contributions presented in the study are included in the article/**Supplementary Materials**. Further inquiries can be directed to the corresponding author.

## Ethics statement

Ethical review and approval were not required for the study on human participants in accordance with the local legislation and institutional requirements. Written informed consent for participation was not required for this study in accordance with the national legislation and the institutional requirements.

## Author contributions

JA, RW, AS and RF contributed to the conception and design of the study. JA performed the experiments and data analysis described in this manuscript. All authors contributed to the article and approved the submitted version 
