# Supplementary material for: Rapid screening and scaled manufacture of immunogenic virus-like particles in a tobacco BY-2 cell-free protein synthesis system
Source: Front Immunol. 2023 Jan 26;14:1088852. doi: 10.3389/fimmu.2023.1088852 (PMC9909599; doi:10.3389/fimmu.2023.1088852)
Supplement: Supplementary file 1 [file DataSheet_1.pdf]

Supplementary Material

1.1 Supplementary Figures

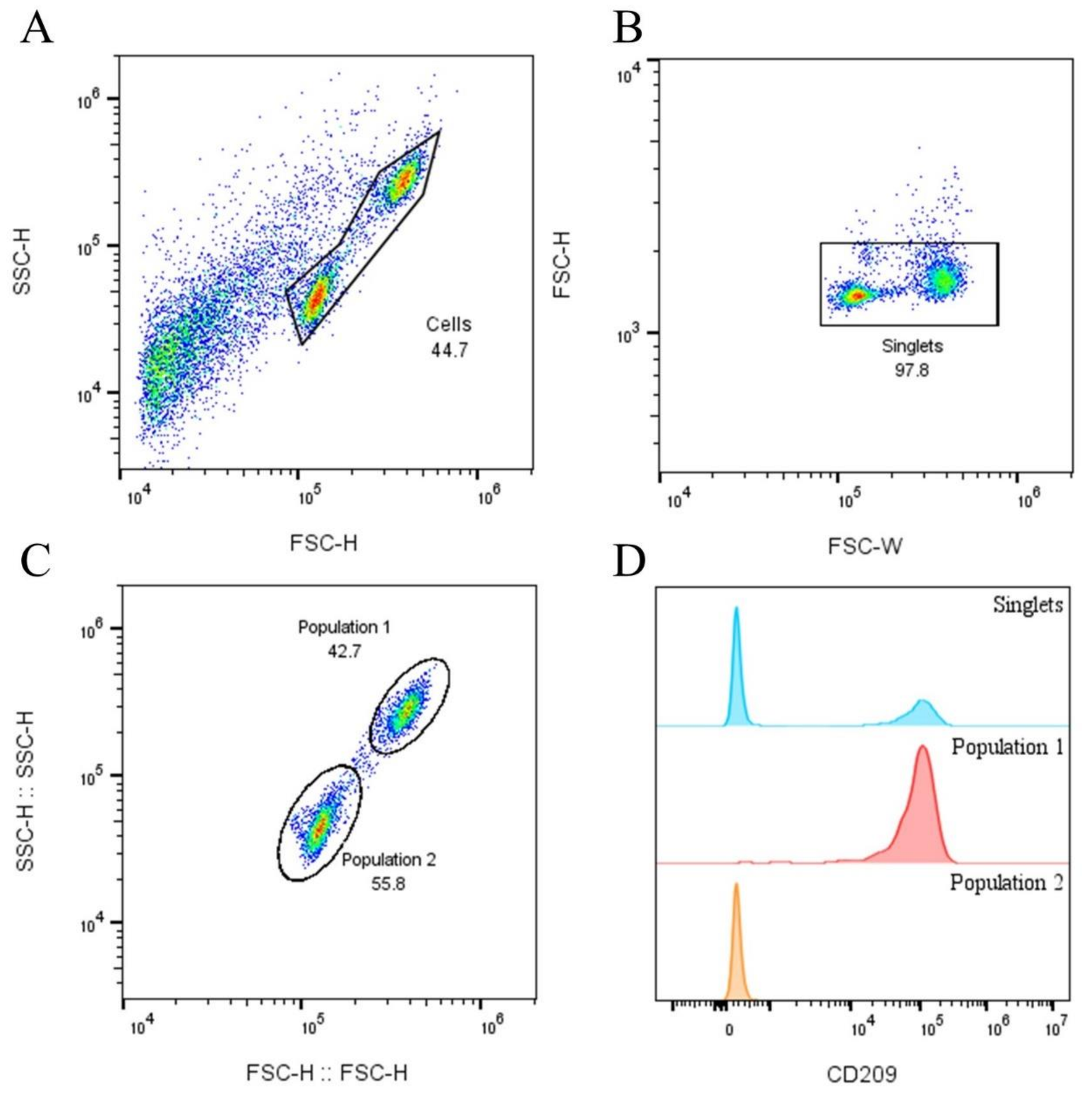

Figure S1. Gating strategy applied for dendritic cell and VLP uptake. Immature Dendritic Cells were differentiated from monocytes isolated from peripheral blood mononuclear cells. Directly after harvest, they were incubated for 2 hours with fluorescently labelled VLP, washed, and labelled with an anti-CD209 (DC-SIGN) Alexa 647-labelled antibody for flow cytometry analysis. **A.** Selection of the two different cell populations in the forward against side scatter plot **B.** Singlet selection in the width versus height plot **C.** Selection of each cell population on the forward against side scatter plot **D.** Histograms of the CD209 marker fluorescence (APC channel) for the singlets, and the two different populations, identifying population 1 as dendritic cells (CD209 positive).

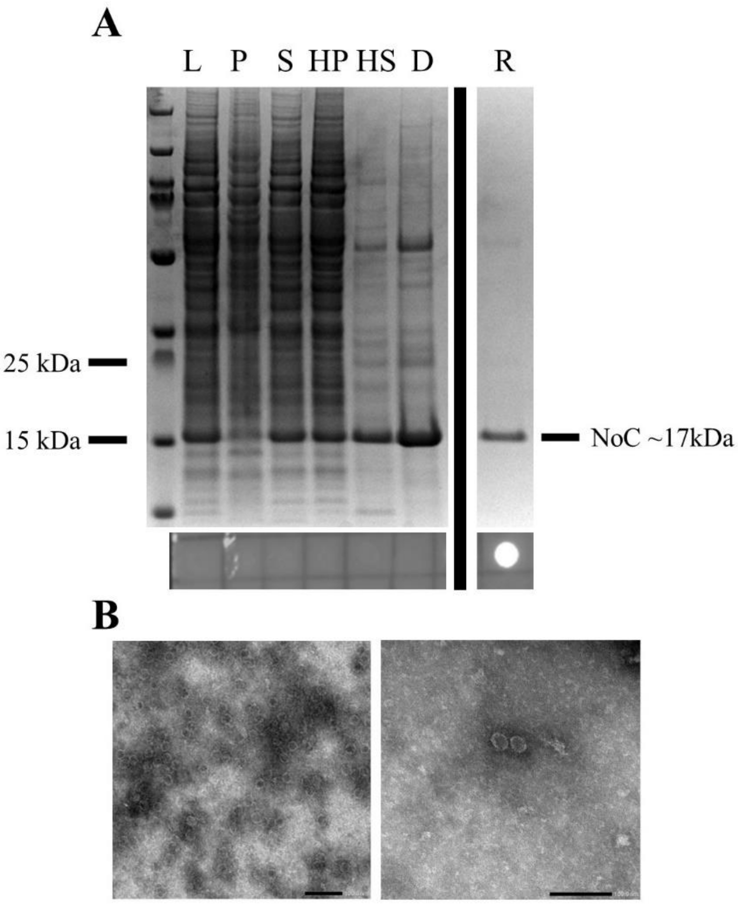

Figure S2. Disassembly and re-assembly of the NoC construct. A. SDS-PAGE gel showing the disassembly and re-assembly process above, and dot blot analysis for the same samples at the bottom. For the dot blot, the conformational antibody 3120 was used, binding only properly assembled particles. B. Transmission Electron Microscopy pictures of re-assembled NoC VLPs. Abbreviations. NoC: HBc core antigen lacking the C-terminal domain; L: lysate after reaction; P: pellet fraction after lysate centrifugation S: supernatant fraction after lysate centrifugation; HP: pellet after heat treatment HS Supernatant after heat treatment D: Disassembled NoC VLP; R: re-assembled NoC VLP.

Table S1 Percentage of live PBMCs 24 hours after stimulation for each donor. Percentage of live cells were measured using a viability staining kit. The average values for the three different stimulation conditions are shown: PBS buffer, Lipopolysaccharide (LPS, 100ng/ml) and Hepatitis B core Virus-like particles (HBc, 10µg/ml). Abbreviations: PBMCs: peripheral blood mononuclear cells.

	Donor 1	Donor 2	Donor 3	Donor 4	Donor 5	Donor 6
PBS	83.43%	82.43%	82.20%	78.50%	83.97%	82.32%
LPS	81.17%	81.50%	83.00%	82.77%	89.89%	88.63%
HBc	80.60%	83.20%	83.63%	87.60%	85.66%	85.88%
